# Joint DNA-based disaster victim identification

**DOI:** 10.1038/s41598-021-93071-5

**Published:** 2021-07-01

**Authors:** Magnus D. Vigeland, Thore Egeland

**Affiliations:** 1 Department of Medical Genetics, University of Oslo, Pb 4956 Nydalen, Oslo Norway; 2 Faculty of Chemistry, Biotechnology and Food Science, Norwegian University of Life Sciences, 1433 Aas, Norway

**Keywords:** Statistics, Computational science, Bioinformatics, Statistical methods, Software

## Abstract

We address computational and statistical aspects of DNA-based identification of victims in the aftermath of disasters. Current methods and software for such identification typically consider each victim individually, leading to suboptimal power of identification and potential inconsistencies in the statistical summary of the evidence. We resolve these problems by performing joint identification of all victims, using the complete genetic data set. Individual identification probabilities, conditional on all available information, are derived from the joint solution in the form of *posterior pairing probabilities*. A closed formula is obtained for the a priori number of possible joint solutions to a given DVI problem. This number increases quickly with the number of victims and missing persons, posing computational challenges for brute force approaches. We address this complexity with a preparatory sequential step aiming to reduce the search space. The examples show that realistic cases are handled efficiently. User-friendly implementations of all methods are provided in the R package **dvir**, freely available on all platforms.

## Introduction

DNA-based *disaster victim identification* (DVI) is a rapidly developing field in forensic genetics, with important applications all around the world. Recent, high-profile cases include the after-math of the 1990-s Balkan conflicts^1^, drowned migrants in Italy^2^, the World Trade Center attack, USA^3^, Thailand tsunami 2004^4^, and the search for missing grandchildren in Argentina^5^.

**Figure 1:**
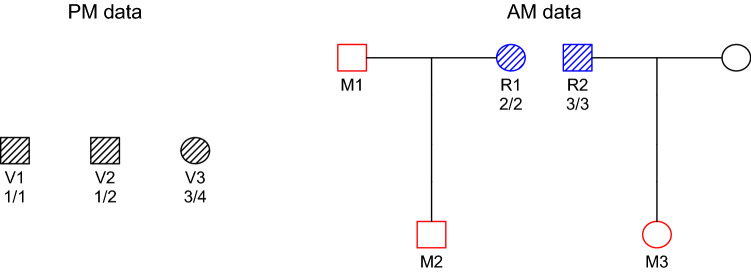
A toy DVI problem. The PM data consists of 3 victim samples to be matched against 3 missing persons (red) belonging to two different families. The AM data contains profiles from the reference individuals $\mathrm {R}_1$ and $\mathrm {R}_2$ (blue), one from each family. Squares and circles represent males and females, respectively. The hatched individuals are typed with a single marker, with genotypes as shown.

In a broader context, DVI involves a variety of data sources and experts from several branches of forensic science, including anthropology, odontology, pathology as well as genetics. The genetic data typically consists of *post mortem* (PM) DNA from victim samples and *ante mortem* (AM) DNA from relatives of the missing persons. Additional data like the sex and age is used if available. Extensive background and general guidelines for handling DVI problems are given in papers^6–8^. In this paper we restrict our attention to computational and statistical aspects of identification cases involving multiple victims, often called *mass identifications* in the literature. A simple example of such a case is shown in Fig. 1.

Current approaches to mass identification typically employ either a (i) *one-to-one*, (ii) *PM-driven*, or (iii) *AM-driven* search strategy^9^. The one-to-one approach simply amounts to comparing each PM profile to each AM reference, looking for evidence of a close relationship. This method is widely used, at least for an initial screening, since easy cases, like direct matches and parent-child, often can be reliably resolved in this way^2,9^. In more complex cases, however, the one-to-one strategy is not sufficient. For a trivial example, observe that this method cannot identify the missing person $\mathrm {M}_1$ in Fig. 1, who is not genetically related to any of the reference individuals.

The PM-driven and AM-driven approaches proceed sequentially, considering one victim (PM-driven) or one family (AM-driven) at the time. We concentrate on the PM-driven in the following. Briefly, the idea is to start with any victim $\mathrm {V}\!$, and to calculate the likelihood ratio (LR) comparing $\mathrm {V}\!$ to each missing person $\mathrm {M}$. The largest LR points to the most likely match for $\mathrm {V}\!$, and a successful identification is declared if this largest LR exceeds a prescribed threshold. Also, if *priors* are specified the LRs can be converted to *posterior probabilities*^10^.

The above description glosses over several important points with potential impact on the output solutions. Detailed descriptions of two possible implementations of the PM-driven sequential approach are given in the Methods section.

**Figure 2:**
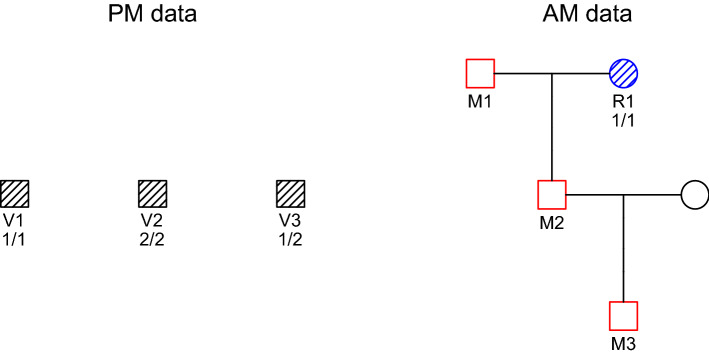
A simple case where sequential approaches fail. Samples from three victims ($\mathrm {V}\!_1, \mathrm {V}\!_2, \mathrm {V}\!_3$) are matched against three missing family members ($\mathrm {M}_1, \mathrm {M}_2, \mathrm {M}_3$), using the grandmother $\mathrm {R}_1$ as reference. Genotypes for a single marker are shown.

While sequential methods may be useful in certain scenarios, they are not optimal from a statistical viewpoint and may lead to ambiguous results. The three approaches (i)–(iii) are all *restricted*, in the sense that they utilise only parts of the data in each step. A simple example of how this may cause missed identifications is given in Fig. 2, where we show that the missing person ($\mathrm {M}_1$) cannot be identified unless the data are considered jointly (see “Results”). The take-home message is that the best match for one individual may obstruct the most likely overall solution.

Another problem with restricted methods is that they may produce inconsistent results. For example, since conclusions are reached independently for each victim (or family), it may happen that a victim is classified as being the most likely member of two different families.

Our goal has been to present methods and implementations that provide consistent solutions to DVI problems, by considering all available data simultaneously. This is achieved by Algorithm 3 in the Methods section, which finds the most likely solution among all possible, while keeping the need for brute force calculations to a minimum.

Decision makers and the legal system often require independent conclusions for each missing person. In response to this, we provide formulas for *posterior pairing probabilities* for each victim - missing person pair.

To the best of our knowledge, no freely available software offer joint DVI computations. The restricted strategies mentioned above are implemented in Familias^11^, but currently allow only one missing person in each family. Commercial software like Bonaparte^12,13^ and DNA View^3^ provide similar functionality, but precise details on the implementation are not publically available.

To rectify this we have developed the R package **dvir**, based on the **ped suite** ecosystem for pedigree analysis in R^14^. The data sets analysed in this paper are included as part of **dvir**, and further examples are given in the documentation. The source code is freely available from https://github.com/thoree/dvir.

## Methods

The starting point of our investigations is a DVI situation involving *s* victim samples, hypothesised to belong to some or all of *m* missing persons (MPs). Identification is done by genetic matching against relatives of the missing persons, using a battery of forensic markers.

In our examples we assume all markers to be independent autosomal markers in Hardy–Weinberg Equilibrium (HWE). However, it should be noted that the overall approach applies very generally; in fact the only requirement is that likelihoods can be calculated. Some extensions are conceptually simple, like mutation modelling or X-chromosomal markers, while others are more challenging, as accommodating linkage disequilibrium. In our R package **dvir**, likelihoods calculations are internally handled with the **pedprobr** package, which is based on the Elston–Stewart algorithm and allows many of these generalisations(^14^, Chapter 5).

We proceed to describe the input data in a bit more detail, and introduce some important notation.

**PM data**: The *post mortem* (PM) samples are denoted $\mathrm {V}\!_1,\dots ,\mathrm {V}\!_s$. We assume throughout that these belong to different individuals. In practice, samples gathered from disaster victims often contain duplicates, requiring a preprocessing step in order to identify and merge these^15^. In our examples we also assume that the sex of each $\mathrm {V}\!_i$ is known, with $s_F$ females and $s_M$ males so that $s_F + s_M = s$. We note that this assumption is not vital to our methods, but helps to narrow the search space.
**AM data**: The *ante mortem* (AM) data consist of one or more reference families, each containing at least one missing person (denoted $\mathrm {M}_1, \mathrm {M}_2, \dots , \mathrm {M}_m$) and at least one genotyped reference member (denoted $\mathrm {R}_1, \mathrm {R}_2, \dots$). Again we assume known sex of all family members. In particular, let $m_F$ and $m_M$ be the number of female and male missing persons, respectively, with $m_F+m_M = m$.

A possible solution, referred to as an *assignment*, to the DVI problem we are addressing, is a one-to-one correspondence between a subset of $\mathscr {V} = \{\mathrm {V}\!_1,\dots ,\mathrm {V}\!_s\}$ and a subset of $\mathscr {M} = \{\mathrm {M}_1,\dots ,\mathrm {M}_m\}$, with the requirement that all identifications are sex consistent. For example, in Fig. 1, a consistent assignment is $\{\mathrm {V}\!_1 = \mathrm {M}_2, \mathrm {V}\!_3 = \mathrm {M}_3\}$. Alternatively, we may write this more compactly as a tuple $(\mathrm {M}_2, *, \mathrm {M}_3)$, whose *i*’th element is the match for $\mathrm {V}\!_i$, or ‘$*$‘ if the assignment does not include a match for $\mathrm {V}\!_i$. In the case of Fig. 1 there are in total 14 assignments, as listed in the first three columns of Table 1.

**Table 1:** The 14 possible assignments for the DVI problem in Fig. 1, ranked according to LR.

	$\mathrm {V}\!_{1}$	$\mathrm {V}\!_{2}$	$\mathrm {V}\!_{3}$	Loglik	LR	Posterior
1	$\mathrm {M}_{1}$	$\mathrm {M}_{2}$	$\mathrm {M}_{3}$	− 16.12	250.00	0.72
2	$\mathrm {M}_{1}$	$\mathrm {M}_{2}$	*	− 17.73	50.00	0.14
3	*	$\mathrm {M}_{2}$	$\mathrm {M}_{3}$	− 18.42	25.00	0.07
4	$\mathrm {M}_{1}$	*	$\mathrm {M}_{3}$	− 20.03	5.00	0.01
5	*	$\mathrm {M}_{1}$	$\mathrm {M}_{3}$	− 20.03	5.00	0.01
6	*	$\mathrm {M}_{2}$	*	− 20.03	5.00	0.01
7	*	*	$\mathrm {M}_{3}$	− 20.03	5.00	0.01
8	$\mathrm {M}_{1}$	*	*	− 21.64	1.00	0.00
9	*	$\mathrm {M}_{1}$	*	− 21.64	1.00	0.00
10	*	*	*	− 21.64	1.00	0.00
11	$\mathrm {M}_{2}$	$\mathrm {M}_{1}$	$\mathrm {M}_{3}$	− Inf	0.00	0.00
12	$\mathrm {M}_{2}$	$\mathrm {M}_{1}$	*	− Inf	0.00	0.00
13	$\mathrm {M}_{2}$	*	$\mathrm {M}_{3}$	− Inf	0.00	0.00
14	$\mathrm {M}_{2}$	*	*	− Inf	0.00	0.00

Note that the empty assignment $(*,*,*)$ is a valid solution, referred to as the *null model* below.

The *likelihood* *L*(*a*) of an assignment *a* is defined as the probability

$$L(a) = P(\text {PM and AM data} \mid a, \Phi ),$$

where the fixed parameters $\Phi$ include the reference pedigrees, marker allele frequencies and mutation models. To simplify the notation we write $L_0$ for the likelihood of the empty assignment, i.e., corresponding to the hypothesis that all victims are unrelated to all the missing persons. Moreover, we define $\mathrm {LR}_{i,j} = L(\mathrm {V}\!_i = \mathrm {M}_j)/L_0$ to be the likelihood ratio of the assignment $\{\mathrm {V}\!_i = \mathrm {M}_j\}$, giving rise to the *pairwise LR matrix*,1

It should be noted that the likelihoods appearing in the definition of $\mathrm {LR}_{i,j}$ involve the complete PM and AM datasets. However, simpler calculations are obtained by considering the reduced DVI problem (PM$_i$, AM$_j$), where PM$_i$ is just $\mathrm {V}\!_i$, and AM$_j$ consists of data from the relatives of $\mathrm {M}_j$. Then it is straightforward to show that

$$\begin{aligned} \mathrm {LR}_{i,j} = \frac{P(\text {PM}_i, \text {AM}_j \mid \mathrm {V}\!_i = \mathrm {M}_j)}{P(\text {PM}_i, \text {AM}_j \mid \mathrm {V}\!_i \text { unrelated to } \mathrm {M}_j)}. \end{aligned}$$

In the simple case shown in Fig. 1, the matrix *B* can be computed by hand. Let us assume that the marker has 10 alleles 1,2, …, 10, with equal frequencies $p_1 =\dots = p_{10} = 1/10$. We then have2

For example, the element $\mathrm {LR}_{2,2}$ is the LR when $\mathrm {V}\!_2 = \mathrm {M}_2$ is tested against the hypothesis that $\mathrm {V}\!_2$ and $\mathrm {M}_2$ are unrelated. This gives $\mathrm {LR}=p_1/(2p_1p_2)=5$. Obviously, convincing LRs cannot be expected in this case, with only a single marker.

The zero elements of *B* correspond to sex-inconsistent pairings or exclusions. Furthermore, we see that the DNA data is uninformative for some of the pairings. The entries $\mathrm {LR}_{1,1} = \mathrm {LR}_{2,1} = 1$ result from the fact that $\mathrm {M}_1$ is not related to either of the reference individuals, and imply that he can never be identified unless $\mathrm {M}_2$ is identified first.

### The number of assignments

Let $\mathscr {A}$ be the set of all sex-consistent assignments for a given DVI problem. The total number of elements, $n = |\mathscr {A}|$, is a good measure of the problem’s size, and may indicate whether a brute force approach is feasible. Consider first the situation where sex is not known neither for victims nor MPs. The total number of assignments is then

3 $$\begin{aligned} \sum _{k=0}^{\min (s, m)} {s\atopwithdelims ()k}{m\atopwithdelims ()k}k!. \end{aligned}$$

The reasoning is as follows: For each *k*, there are $$\left( {\begin{array}{c}s\\ k\end{array}}\right) $$ different subsets of *k* victims. Each of these can be assigned to $$\left( {\begin{array}{c}m\\ k\end{array}}\right) $$ different subsets of the *m* missing persons. Finally, each assignment can be shuffled in *k*! ways.

When the sexes are known, formula (3) applies to females and males independently, and the total number becomes

4 $$\begin{aligned} n = n(s_F, s_M, m_F, m_M) = \left[ \sum _{k=0}^{\min (s_F,m_F)} {s_F\atopwithdelims ()k}{m_F\atopwithdelims ()k}k! \right] \left[ \sum _{k=0}^{\min (s_M,m_M)} {s_M\atopwithdelims ()k}{m_M\atopwithdelims ()k}k! \right] . \end{aligned}$$

Unsurprisingly, *n* increases rapidly with the number of victims and missing persons, but depends strongly on the distribution of sexes. To illustrate, Table 2 tabulates the number of assignments with 8 victims and 5 MPs, for all combinations of males/females. The total of 19081 assignments when all victims and MPs have the same sex, is considerably higher than in all other cases.

**Table 2:** The number of sex-consistent assignments in a DVI case with 5 victims and 8 MPs.

$m_F$	$s_F$					
	0	1	2	3	4	5
0	19,081	3393	529	73	9	1
1	9276	3922	1074	228	40	6
2	4051	3135	1603	559	147	31
3	1546	2004	1768	1054	438	136
4	501	1045	1533	1533	1045	501
5	136	438	1054	1768	2004	1546
6	31	147	559	1603	3135	4051
7	6	40	228	1074	3922	9276
8	1	9	73	529	3393	19,081

The variables $s_F$ and $m_F$ denote the number of female victims and MPs, respectively.

### Sequential approaches

Here we describe two natural implementations of the PM-driven search strategy. As alluded to in the introduction this sequential approach is suboptimal in several ways, but it may be the best option in very large-scale applications. The motivation for including these algorithms here is to expose and clarify implementational details, and to serve as reference for the novel methods described later.

#### Algorithm 1: sequential (without updates)

*Input* A DVI problem; a threshold $T > 1$.

*Output* A proposed solution to the DVI case in the form of an assignment *a*. (In case of ties, more than one assignment may result.)

*Procedure*

(i) Compute the pairwise LR matrix *B*.
(ii) If all elements of *B* are below *T*, then stop. Otherwise, let $\mathrm {LR}_{i,j}$ be the maximal element of *B* and store the identification $\mathrm {V}\!_i = \mathrm {M}_j$. If there are multiple maximal elements, branch off and proceed with one at a time.
(iii) Update *B* by deleting the row and column corresponding to $\mathrm {LR}_{i,j}$.
(iv) Repeat steps (ii) - (iii) until the procedure stops.

To illustrate Algorithm 1, consider our running example from Fig. 1, for which the pairwise LR matrix was given in (2). If $T> 5$, no identifications are made. For any $T\le 5$, the above algorithm identifies $\mathrm {V}\!_2 = \mathrm {M}_2$ and $\mathrm {V}\!_3 = \mathrm {M}_3$ (both with $\mathrm {LR}= 5$), after which the procedure stops. Hence the reported solution is $(*, \mathrm {M}_2, \mathrm {M}_3)$. As remarked earlier, $\mathrm {M}_1$ cannot be identified with this approach.

The next algorithm is a refinement of Algorithm 1, with the crucial difference that the LR matrix is now recomputed in each step.

#### Algorithm 2: sequential (with updates)

*Input* A DVI problem; a threshold $T > 1$.

*Output* A proposed solution to the DVI problem in the form of an assignment *a*. (In case of ties, more than one assignment may result.)

*Procedure* As Algorithm 1, but where step (iii) is replaced with the following:

(iii) Update *B* by deleting the row and column corresponding to $\mathrm {LR}_{i,j}$, and recomputing the remaining LR values conditional on all previous identifications.

When this strategy is applied to the example in Fig. 1, the sequence of updated $\mathrm {LR}$ matrices becomes as follows:5 In both cases, the identified solution is $(\mathrm {M}_1, \mathrm {M}_2, \mathrm {M}_3)$.

### The joint approach

We now consider the possibility (and feasibility) of *joint* identification of the victims. Among the list $\mathscr {A}$ of all *a priori* possible assignments, we seek the one that maximises the overall likelihood: An assignment $a^*$ is an optimal solution if $L(a^*) \ge L(a)$ for all $a\in \mathscr {A}$. In smaller cases where $|\mathscr {A}|$ [as given by formula (4)] is manageable , this may be solved by brute force, i.e., by calculating the likelihood of each assignment, and sorting them in descending order.

Applying this to our running example in Fig. 1, Table 1 lists the likelihoods of all 14 possibilities. It shows that assignment $(\mathrm {M}_1, \mathrm {M}_2, \mathrm {M}_3)$ is a clear winner, five times more likely than the runner-up. In this case all calculations may be done manually. For example, under the null model $(*,*,*)$ all five genotyped individuals are unrelated, giving the likelihood $L_0 = 4\cdot (0.1)^{10}$ and (natural) log-likelihood $\log (L_0) = -21.64$ as shown in line 10 of Table 1.

### Combined approach

In larger cases the number of possible assignments may be prohibitive for brute force calculations. In this case, we propose the combination approach described below. In brief, the idea is to first use a modified version of Algorithm 2 in order to find *undisputed* pairings, and then use brute force on the remaining problem. A pairing $(\mathrm {V}\!_i, \mathrm {M}_j)$ is said to be undisputed if its pairwise-search LR reaches the given threshold *T*, while all other LR values involving $\mathrm {V}\!_i$ or $\mathrm {M}_j$ are small.

#### Algorithm 3: undisputed + joint

*Input* A DVI problem; a threshold $T > 1$.

*Output* A list of assignments, ranked by likelihood.

*Procedure*

Step 1 Sequential.
  (i) Compute the pairwise LR matrix *B*.
  (ii) Identify all undisputed pairings $\mathrm {V}\!_i = \mathrm {M}_j$, characterised by $\mathrm {LR}_{i,j} \ge T$ while all other entries in the same row and column are $\le 1$. If no such elements are found, the procedure stops.
  (iii) Update *B* by deleting rows and columns corresponding to undisputed pairings and recomputing the remaining $\mathrm {LR}$s conditional on the same.
  (iv) Repeat steps (ii)–(iii) until the procedure stops.
Step 2 Joint.
  (i) Create a list $\mathscr {A}$ of sex-consistent assignments involving the remaining individuals.
  (ii) Remove from $\mathscr {A}$ all impossible assignments, i.e., corresponding to zeroes in the updated LR matrix *B*.
  (iii) Compute the likelihood of the remaining assignments in $\mathscr {A}$, conditional on the undisputed findings, and rank the output.

The combined approach may still fail if there are too many assignments to consider in Step 2. A practical solution is then to reduce the threshold *T* so that the number of undisputed matches is increased, leaving fewer remaining individuals for the joint analysis.

### Posterior pairing probabilities

In this section we derive the *posterior pairing probabilities* $q_{i,j} = P(\mathrm {V}\!_i = \mathrm {M}_j \mid D)$ for $i = 1 \dotsc , s$ and $j = 1, \dotsc , m$, where *D* denotes the PM and AM data. For each victim $\mathrm {V}\!_i$ we also compute the *posterior non-pairing probability*, $q_{i,*} = P(\mathrm {V}\!_i = * \mid D)$, i.e., the probability that $\mathrm {V}\!_i$ does not match any of the missing persons.

These probabilities are relevant since decisions often need to be made for each individual independently. Importantly, this approach opens for incorporating non-DNA information via a prior distribution. For any assignment $a \in \mathscr {A}$, let $\pi (a)$ denote the prior probability of *a*.

For a given pair $(\mathrm {V}\!_i, \mathrm {M}_j)$, let $\mathscr {A}_{i,j}$ denote the subset of $\mathscr {A}$ consisting of all assignments containing the pairing $\mathrm {V}\!_i = \mathrm {M}_j$. Bayes’ theorem then gives

6 $$\begin{aligned} q_{i,j} = P(\mathrm {V}\!_i = \mathrm {M}_j \mid D) =\frac{\sum _{a \in \mathscr {A}_{i,j}} L(a) \pi (a)}{\sum _{a \in \mathscr {A}} L(a) \pi (a)}, \end{aligned}$$

where, as before, *L*(*a*) is the likelihood of *a*. Often a flat prior $\pi (a)=1/|\mathscr {A}|$ is used, in which case (6) can be written in terms of likelihood ratios:

7 $$\begin{aligned} q_{i,j} =\frac{\sum _{a \in \mathscr {A}_{i,j}} \mathrm {LR}_a}{\sum _{a \in \mathscr {A}} \mathrm {LR}_a}. \end{aligned}$$

Here, $\mathrm {LR}_a$ denotes the likelihood ratio comparing *a* to the empty (null) assignment.

The posterior non-pairing probabilities are computed similarly: If $\mathscr {A}_{i,*}$ denotes the set of assignments with no match for $\mathrm {V}\!_i$, we have

8 $$\begin{aligned} q_{i,*} = P(\mathrm {V}\!_i = * \mid D) =\frac{\sum _{a \in \mathscr {A}_{i,*}} L(a) \pi (a)}{\sum _{a \in \mathscr {A}} L(a) \pi (a)} = \frac{\sum _{a \in \mathscr {A}_{i,*}} \mathrm {LR}_a}{\sum _{a \in \mathscr {A}} \mathrm {LR}_a}, \end{aligned}$$

where the latter equality assumes a flat prior.

For our running example in Fig. 1, the posterior probabilities with a flat prior are given in Table 3. Note that these probabilities are directly calculable from the LR column of Table 1, which provides the likelihood ratios required by formulas (7) and (8). For example, the top left entry is

$$\begin{aligned} q_{1,1} = \frac{250 + 50 + 5 + 1}{250 + 50 + 25 + 5 + 5 + 5 + 5 + 1 + 1 + 1} \approx 0.88. \end{aligned}$$

**Table 3:** Posterior pairing probabilities for the toy example in Fig. 1.

	$\mathrm {M}_{1}$	$\mathrm {M}_{2}$	$\mathrm {M}_{3}$	$*$
$\mathrm {V}\!_{1}$	0.88	0.00	0.00	0.12
$\mathrm {V}\!_{2}$	0.02	0.95	0.00	0.03
$\mathrm {V}\!_{3}$	0.00	0.00	0.83	0.17

It is reasonable to conclude that $\mathrm {V}\!_i=\mathrm {M}_j$ if $q_{i,j} > \alpha$ for some $\alpha$ close to 1, say $\alpha =0.99$. A less stringent threshold $\alpha = 0.5$ could be used if the objective is only to find the most likely match. Importantly, as long as $\alpha > 0.5$ any pairings obtained in this way are *consistent*, in the sense that two victims cannot be paired with the same missing person. To show this, let $\mathrm {V}\!_i$ and $\mathrm {V}\!_{i'}$ denote two different victims. Then for any *j* the sets $\mathscr {A}_{i,j}$ and $\mathscr {A}_{i', j}$ are disjoint, so that

$$\begin{aligned} q_{i,j} + q_{i',j} = P(\mathrm {V}\!_i=\mathrm {M}_j\mid D)+P(\mathrm {V}\!_{i'}=\mathrm {M}_j\mid D)=\sum _{a \in \mathscr {A}_{i,j}}P(a\mid D)+\sum _{a \in \mathscr {A}_{i',j}}P(a\mid D) \le \sum _{a \in \mathscr {A}}P(a\mid D)=1. \end{aligned}$$

This implies that $q_{i,j}$ and $q_{i',j}$ cannot both exceed 0.5.

## Results

### A comparison of methods

The purpose of the example is to illustrate that the joint approach may succeed in cases where the sequential methods fail.

Consider the DVI problem shown in Fig. 2, where the genotypes correspond to a marker with alleles 1, 2, and 3, with frequencies 0.05, 0.05 and 0.9 respectively. (The precise values of these frequencies are not important.) Given this information, the pairwise LR matrix is found to be9

As a first observation we note that the column of 1’s corresponding to $\mathrm {M}_1$ implies that $\mathrm {M}_1$ cannot identified by any method which uses data from only one victim at a time, such as Algorithm 1.

Next we consider the more reasonable Algorithm 2, which updates *B* after each new pairing. Clearly, since $\mathrm {LR}_{1,2} = 20$ is the highest entry, the procedure starts by identifying $\mathrm {V}\!_1 = \mathrm {M}_2$. But this means that $\mathrm {M}_2$ has genotype 1/1, which effectively blocks $\mathrm {V}\!_2$ (who is 2/2) from being identified as $\mathrm {M}_1$ or $\mathrm {M}_3$. In full detail, the sequence of updated LR matrices becomes as follows:10 We conclude that Algorithm 2 produces two equally likely assignments, $(\mathrm {M}_2, *, \mathrm {M}_1)$ and $(\mathrm {M}_2, *, \mathrm {M}_3)$.

By contrast, Table 4 shows that the optimal solution, when all the data is considered jointly, is the assignment $(\mathrm {M}_3, \mathrm {M}_1, \mathrm {M}_2)$. In fact, this is $2,000/200=10$ times more likely than either of the solutions found by the sequential method above. Table 5 lists the posterior pairing probabilities under a flat prior.

**Table 4:** The most likely assignments in Fig. 2.

	$\mathrm {V}\!_{1}$	$\mathrm {V}\!_{2}$	$\mathrm {V}\!_{3}$	Loglik	LR	Posterior
1	$\mathrm {M}_{3}$	$\mathrm {M}_{1}$	$\mathrm {M}_{2}$	-15.67	2,000.00	0.69
2	$\mathrm {M}_{2}$	$*$	$\mathrm {M}_{1}$	-17.97	200.00	0.07
3	$\mathrm {M}_{2}$	$*$	$\mathrm {M}_{3}$	-17.97	200.00	0.07
4	$*$	$\mathrm {M}_{1}$	$\mathrm {M}_{2}$	-17.97	200.00	0.07
5	$\mathrm {M}_{3}$	$*$	$\mathrm {M}_{2}$	-18.67	100.00	0.03
6	$*$	$\mathrm {M}_{3}$	$\mathrm {M}_{2}$	-18.67	100.00	0.03

**Table 5:** Posterior pairing probabilities for the case in Fig. 2.

	$\mathrm {M}_{1}$	$\mathrm {M}_{2}$	$\mathrm {M}_{3}$	$*$
$\mathrm {V}\!_{1}$	0.004	0.145	**0.736**	0.115
$\mathrm {V}\!_{2}$	**0.766**	0.000	0.036	0.198
$\mathrm {V}\!_{3}$	0.076	**0.831**	0.078	0.015

Values exceeding 0.5 are shown in bold.

**Figure 3:**
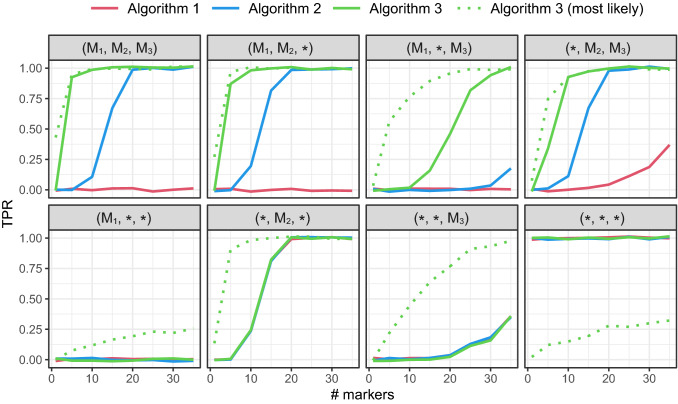
A comparison of the true positive rates (TPR) of different DVI algorithms. Each point is the result of 500 simulations conditional on the assignment indicated in the panel title. For instance, the assignment $( *, \mathrm {M}_2, \mathrm {M}_3)$ in the top right panel signifies that $\mathrm {V}\!_1$ is unidentified, while $\mathrm {V}\!_2=\mathrm {M}_2$ and $\mathrm {V}\!_3=\mathrm {M}_3$. The *x*-axis indicates the number of markers used, in the order listed in the database NorwegianFrequencies (see main text). A slight vertical jitter was applied to the points in order to increase visibility.

In order to investigate the practical relevance of joint identification, we conducted a series of simulation experiments based on standard set of forensic markers. After all, the genotypes in Fig. 2 were particularly chosen so as to illustrate the effect, and with multiple markers one might expect such anomalies to be drowned. Unfortunately, this is not the case. Figure 3 compares how the true positive rates (TPR) of Algorithms 1–3 vary with the number of markers, depending on the true solution as indicated in the title of each panel. In each case, 500 sets of DNA profiles were simulated for the set of 35 autosomal markers comprising the database NorwegianFrequencies available and documented in the R package **forrel**. The simulations were performed with the **forrel** function profileSim. All subsequent identifications used LR threshold $T=10,000$ (for Algorithm 3, the threshold applied to the highest joint LR compared with the null). In addition to the three algorithms previously described, we included the TPR of the *most likely* solution reported by Algorithm 3, whether or not its LR exceeded *T*.

Figure 3 reveals that for this particular DVI problem, the joint method (Algorithm 3) has a TPR near 1 already with 5 markers, while the best sequential (Algorithm 2) needs 20 markers to reach the same. Overall, Algorithm 3 clearly outperforms the others in all cases shown in the top row of Fig. 3. Moreover, it is the only method to reliably reach a conclusion when the true assignment is $(\mathrm {M}_1, *, \mathrm {M}_3)$.

### Case study 1: plane crash

In this and the next section we demonstrate the adequacy of joint identification in real-life scenarios, by analysing two realistic DVI datasets. The first case is based on a simulated plane crash, and features multiple reference families with a single missing individual. The second case involves a large pedigree with many missing members.

**Figure 4:**
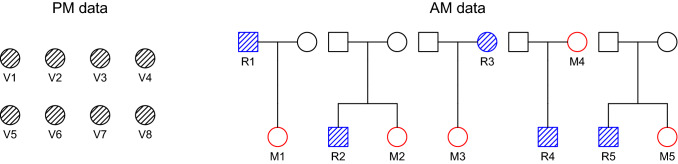
The plane crash scenario. Eight victims are to be matched against five reference families. Each family has one missing person and one reference individual.

Figure 4 lays out the components of a DVI problem in the aftermath of a plane crash. DNA profiles with 15 standard forensic markers are available from 8 victims and 5 reference families. The complete dataset is included in the R package **dvir** under the name planecrash. Further information, including marker names, allele frequencies and simulation details can be found in the package documentation.

**Table 6:** Pairwise LRs for the plane crash example.

	$\mathrm {M}_{1}$	$\mathrm {M}_{2}$	$\mathrm {M}_{3}$	$\mathrm {M}_{4}$	$\mathrm {M}_{5}$
$\mathrm {V}\!_{1}$	9.29e+02	9.03e−04			2.77e−01
$\mathrm {V}\!_{2}$		6.75e−02	**6.79e+04**		6.66e−02
$\mathrm {V}\!_{3}$		1.03e−04			3.82e−03
$\mathrm {V}\!_{4}$		3.78e−05			**3.19e+07**
$\mathrm {V}\!_{5}$		9.62e−04			3.92e−03
$\mathrm {V}\!_{6}$		**1.08e+06**			1.29e−05
$\mathrm {V}\!_{7}$		5.90e−04			1.90e−01
$\mathrm {V}\!_{8}$		1.91e−04			2.72e−01

Only nonzero elements are shown; entries reaching the threshold $T=10,000$ are highlighted.

In accordance with Algorithm 3, we start by computing the pairwise LR values, with the results given in Table 6. We observe that the identifications $\mathrm {V}\!_2 = \mathrm {M}_3$, $\mathrm {V}\!_4 = \mathrm {M}_5$ and $\mathrm {V}\!_6 = \mathrm {M}_2$ are undisputed (in the sense defined in Algorithm 3), when applying the threshold $T=10,000$. In addition, $\mathrm {V}\!_1 = \mathrm {M}_1$ also has a high $\mathrm {LR}$, but does not reach the threshold. Based on these observations we anticipate the solution $(\mathrm {M}_1, \mathrm {M}_3, *, \mathrm {M}_5, *, \mathrm {M}_2, *, *)$. Indeed, this assignment is the optimal solution found in the joint analysis, presented in Table 7. It is noteworthy that the many impossible and undisputed pairings in the pairwise LR matrix (Table 6) leave only two assignments for consideration in the joint step. Hence the computational cost of this step is virtually ignorable.

**Table 7:** Results of joint analysis of the plane crash example, without mutation modelling.

	$\mathrm {V}\!_{1}$	$\mathrm {V}\!_{2}$	$\mathrm {V}\!_{3}$	$\mathrm {V}\!_{4}$	$\mathrm {V}\!_{5}$	$\mathrm {V}\!_{6}$	$\mathrm {V}\!_{7}$	$\mathrm {V}\!_{8}$	Loglik	LR	Posterior
1	$\mathrm {M}_{1}$	$\mathrm {M}_{3}$	$*$	$\mathrm {M}_{5}$	$*$	$\mathrm {M}_{2}$	$*$	$*$	− 562.80	2.17e+21	0.999
2	$*$	$\mathrm {M}_{3}$	$*$	$\mathrm {M}_{5}$	$*$	$\mathrm {M}_{2}$	$*$	$*$	− 569.64	2.34e+18	0.001

Next we introduce a mutation model, motivated by the possibility that a mutation may explain the lack of identification for $\mathrm {M}_4$. In fact, an examination of the data reveals that $\mathrm {V}\!_3$ and $\mathrm {R}_4$ share alleles at all but one marker, suggesting that these may have a parent-child relationship.

We use a proportional model with mutation rate 0.001^16^. The choice of model is not significant here, but we note that the property of stationarity enjoyed by proportional models facilitates validation against other implementations. Under this model, the pairwise LR for $\mathrm {V}\!_3 = \mathrm {M}_4$ changes from 0 to 249. A joint analysis produces the top list shown in Table 8. We see that the identification $\mathrm {V}\!_3 = \mathrm {M}_4$ is now convincingly included in the most likely assignment. This observation is reinforced by the posterior pairing probabilities given in Table 9, calculated with a flat prior of $\pi (a)=1/19081$.

**Table 8:** The most likely assignments in the plane crash example, when mutations are modeled.

	$\mathrm {V}\!_{1}$	$\mathrm {V}\!_{2}$	$\mathrm {V}\!_{3}$	$\mathrm {V}\!_{4}$	$\mathrm {V}\!_{5}$	$\mathrm {V}\!_{6}$	$\mathrm {V}\!_{7}$	$\mathrm {V}\!_{8}$	Loglik	LR	Posterior
1	$\mathrm {M}_{1}$	$\mathrm {M}_{3}$	$\mathrm {M}_{4}$	$\mathrm {M}_{5}$	$*$	$\mathrm {M}_{2}$	$*$	$*$	− 557.31	5.27e+23	0.995
2	$\mathrm {M}_{1}$	$\mathrm {M}_{3}$	$*$	$\mathrm {M}_{5}$	$*$	$\mathrm {M}_{2}$	$*$	$*$	− 562.83	2.11e+21	0.004
3	$*$	$\mathrm {M}_{3}$	$\mathrm {M}_{4}$	$\mathrm {M}_{5}$	$*$	$\mathrm {M}_{2}$	$*$	$*$	− 564.14	5.69e+20	0.001
4	$*$	$\mathrm {M}_{3}$	$\mathrm {M}_{4}$	$\mathrm {M}_{5}$	$\mathrm {M}_{1}$	$\mathrm {M}_{2}$	$*$	$*$	− 566.54	5.18e+19	0.000
5	$\mathrm {M}_{1}$	$*$	$\mathrm {M}_{4}$	$\mathrm {M}_{5}$	$*$	$\mathrm {M}_{2}$	$\mathrm {M}_{3}$	$*$	− 566.82	3.89e+19	0.000

**Table 9:** Posterior pairing probabilities in the plane crash example, calculated using a flat prior and a proportional mutational model with rate 0.001.

	$\mathrm {M}_{1}$	$\mathrm {M}_{2}$	$\mathrm {M}_{3}$	$\mathrm {M}_{4}$	$\mathrm {M}_{5}$	$*$
$\mathrm {V}\!_{1}$	0.999					0.001
$\mathrm {V}\!_{2}$			1.000			
$\mathrm {V}\!_{3}$				0.996		0.004
$\mathrm {V}\!_{4}$					1.000	
$\mathrm {V}\!_{5}$						1.000
$\mathrm {V}\!_{6}$		1.000				
$\mathrm {V}\!_{7}$						1.000
$\mathrm {V}\!_{8}$						1.000

### Case study 2: a large reference family

**Figure 5:**
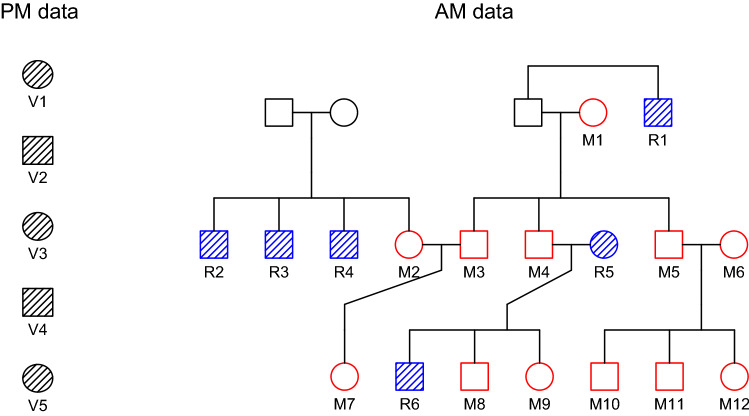
A large reference family with twelve missing individuals.

Our second main example involves the pedigree in Fig. 5, featuring twelve missing individuals ($\mathrm {M}_1, \dots , \mathrm {M}_{12}$) and six typed references ($\mathrm {R}_1, \dots , \mathrm {R}_{6}$). Five victim samples ($\mathrm {V}\!_1, \dots , \mathrm {V}\!_5$) are to be matched against these. The case is based on an example from a workshop organized by the International Society for Forensic Genetics (ISFG)^17^, but we have adapted the pedigree slightly for notational consistency and simulated new marker data. The simulations were performed using 13 CODIS markers, assuming that the true solution is the assignment $(\mathrm {M}_6, \mathrm {M}_{10}, \mathrm {M}_{12}, \mathrm {M}_8, \mathrm {M}_1)$. Further details, including marker names and allele frequencies, are provided in the documentation of the **dvir** dataset icmp.

**Table 10:** Pairwise LRs in case study 2. Values below 0.001 are not shown.

	$\mathrm {M}_{1}$	$\mathrm {M}_{2}$	$\mathrm {M}_{3}$	$\mathrm {M}_{4}$	$\mathrm {M}_{5}$	$\mathrm {M}_{6}$	$\mathrm {M}_{7}$	$\mathrm {M}_{8}$	$\mathrm {M}_{9}$	$\mathrm {M}_{10}$	$\mathrm {M}_{11}$	$\mathrm {M}_{12}$
$\mathrm {V}\!_{1}$	0.0647					1						0.164
$\mathrm {V}\!_{2}$			0.152		0.152					1.25	1.25	
$\mathrm {V}\!_{3}$	0.124					1	0.0156					2.27
$\mathrm {V}\!_{4}$			0.792		0.792			2.85e+06		3.08	3.08	
$\mathrm {V}\!_{5}$	17.5					1	0.00666					2.83

In this case, the pairwise LR matrix (Table 10) shows that there are no undisputed pairings (with $T=10,000$). Admittedly, the pairing $\mathrm {V}\!_4 = \mathrm {M}_8$ has a high $\mathrm {LR}$ at $2.85 \cdot 10^6$, but it is disputed (in the sense of Step 1 - (ii) of Algorithm 3) by $\mathrm {LR}_{4,10}$ and $\mathrm {LR}_{4,11}$ which both exceed 1. (Relaxations of this step are considered in the Discussion.) Nevertheless, the many zeroes in Table 10 lead to a substantial reduction in the space of assignments, manifested in Step 2 - (ii) of Algorithm 3. More precisely, the *a priori* 9847 assignments given by equation (4) (with $s_F = 3, s_M =2, m_F = m_M = 6$) is reduced to 1898 after removal of impossible pairings. Joint analysis of these assignments took $\sim \!15$ seconds on a standard laptop, and resulted in the top list presented in Table 11.

**Table 11:** The five most likely assignments in case study 2.

	$\mathrm {V}\!_{1}$	$\mathrm {V}\!_{2}$	$\mathrm {V}\!_{3}$	$\mathrm {V}\!_{4}$	$\mathrm {V}\!_{5}$	Loglik	LR	Posterior
1	$\mathrm {M}_{6}$	$\mathrm {M}_{10}$	$\mathrm {M}_{12}$	$\mathrm {M}_{8}$	$\mathrm {M}_{1}$	− 312.98	1.14e+24	0.50
2	$\mathrm {M}_{6}$	$\mathrm {M}_{11}$	$\mathrm {M}_{12}$	$\mathrm {M}_{8}$	$\mathrm {M}_{1}$	− 312.98	1.14e+24	0.50
3	$\mathrm {M}_{6}$	$\mathrm {M}_{10}$	$\mathrm {M}_{12}$	$\mathrm {M}_{8}$	$\mathrm {M}_{7}$	− 327.16	7.86e+17	0.00
4	$\mathrm {M}_{6}$	$\mathrm {M}_{11}$	$\mathrm {M}_{12}$	$\mathrm {M}_{8}$	$\mathrm {M}_{7}$	− 327.16	7.86e+17	0.00
5	$\mathrm {M}_{6}$	$*$	$\mathrm {M}_{12}$	$\mathrm {M}_{8}$	$\mathrm {M}_{1}$	− 327.74	4.40e+17	0.00

Note that two assignments tie for the best solutions, differing in their identification of victim $\mathrm {V}\!_2$. This reflects the fact, deducible from Fig. 5, that the pairings $\{\mathrm {V}\!_2=\mathrm {M}_{10}\}$ and $\{\mathrm {V}\!_2=\mathrm {M}_{11}\}$ cannot be distinguished based on DNA data. The posterior pairing probabilities with a flat prior are given in Table 12.

**Table 12:** Posterior pairing probabilities in Case Study 2. Numbers less than 0.001 are not shown.

	$\mathrm {M}_{1}$	$\mathrm {M}_{2}$	$\mathrm {M}_{3}$	$\mathrm {M}_{4}$	$\mathrm {M}_{5}$	$\mathrm {M}_{6}$	$\mathrm {M}_{7}$	$\mathrm {M}_{8}$	$\mathrm {M}_{9}$	$\mathrm {M}_{10}$	$\mathrm {M}_{11}$	$\mathrm {M}_{12}$	$*$
$\mathrm {V}\!_{1}$						1.000							
$\mathrm {V}\!_{2}$										0.500	0.500		
$\mathrm {V}\!_{3}$												1.000	
$\mathrm {V}\!_{4}$								1.000					
$\mathrm {V}\!_{5}$	1.000												

## Discussion

The main contribution of this paper is to show that joint identification generally outperforms sequential DVI methods, and that careful implementation makes the joint approach computationally feasible even in fairly large cases.

From a computational point of view, DVI applications can be viewed as a series of kinship tests. For general issues concerning kinship testing, like the assumptions of Hardy-Weinberg equilibrium and independence of markers, we therefore refer to the rich literature on this subject^18^. Problems related to poor quality of DNA are also widely discussed in the forensic literature^19^. In the following we restrict the discussion to aspects that are particular to the methods of this paper.

The main arguments favouring the joint approach are of principal nature. From a statistical point of view a joint dataset should, by default, be analysed jointly if practically possible. In particular, this avoids inconsistent solutions that may emerge when splitting a joint problem into separate subproblems. However, there are also other important benefits, connected with multiple testing and statistical power. The previously published sequential approaches typically involve many separate tests using different parts of the data. This makes power calculation difficult, if not impossible. In principle one could control for the number of tests performed by increasing the LR threshold, resembling classical post-hoc adjustments in multiple significance testing, but this approach is rarely practical in DVI contexts. The power of our joint approach is not affected by multiple testing issues in the same way, since the problem is not split into many separate tests.

The limiting factor for the utility of joint DVI is the computational burden. Table 2 clearly illustrates the rapid growth of the a priori number of assignments, and it is not difficult to construct problems beyond the current reach. On the other hand, surprisingly large problems may still be manageable if a sufficient number of undisputed matches are found in the first step.

The algorithms we have presented can be modified or tuned in various ways. As in many similar applications, the most important parameter is arguably the $\mathrm {LR}$ threshold *T*. For a general discussion we refer to the established literature^20^, also in connection with familial searching^21^. In the context of DVI we expect the value of *T* to depend both on the particular protocol and external factors. Simulation experiments like the one summarised in Fig. 3 may provide guidance when deciding the threshold.

We mention one potential modification, which may have a significant impact on the run-time of Algorithm 3. Recall that Step 1(ii) of this algorithm used the pairwise $\mathrm {LR}$ matrix to identify undisputed pairings $\mathrm {V}\!_i = \mathrm {M}_j$, characterised by

$\mathrm {LR}_{i,j} \ge T$ while all other entries in the same row and column are $\le 1$.

The last part of this criterion may be relaxed, for instance by increasing the final limit 1 to $\mathrm {LR}_{i,j}/T$. The effect of this change is easily seen in Case Study 2, specifically Table 10, where the pairing $\mathrm {V}\!_4 = \mathrm {M}_6$ would now be classified as undisputed.

For simplicity we used autosomal markers in our examples, but there are no methodological obstructions to including mtDNA, X or Y markers in joint DVI computations. In fact, our implementation in the **dvir** package already supports X-chromosomal markers. As previous authors have noted, it is not obvious how evidence from different types of markers should be reported, and opinions differ^22^.

A well-known challenge in forensic genetics is that LR calculations are sensitive to misspecified allele frequencies. In some DVI cases it may therefore be difficult to decide on an appropriate frequency database, particularly if the individuals originate from different populations. This problem has previously been addressed in the context of familial searching^23^. A practical approach is to do *ad hoc* sensitivity calculations. If the overall conclusions remain unchanged with different databases, this strengthens the confidence in the results. As a general comment we note that most autosomal forensic markers have been specifically selected for their relatively stable allele frequencies across populations, while this to a lesser extent holds for mtDNA, X or Y markers.

The question of how the statistical evidence should be reported in identification cases is difficult and lacks general consensus. Although there is a tradition of specifying priors and reporting posterior probabilities in addition to likelihood ratios^10^, our view is that specifying priors should be left to the decision makers. This is supported by ISFG recommendations^6^, whose point 11 includes:

In DVI work, DNA statistics are best represented as likelihood ratios that permit DNA results to be combined among multiple genetic systems or with other non-DNA evidence.

Nevertheless, we have included in several tables the posteriors with a flat prior, for reference. In real cases, information beyond the DNA data can be reflected by the prior.

Another point related to reporting is the choice of *reference*, i.e., the hypothesis in the denominator of the likelihood ratio. In our examples we have chosen to compare with the null, i.e., no relations between the victims and the missing persons, but it is not obvious that this is always the best choice. For instance, in order to communicate the uniqueness of the solution, a viable alternative is to compare the best solution to the second best. We suggest reporting the identification defined by the optimal assignment if its LR compared to closest contender exceeds a threshold, say 10,000. Clearly, this ensures that the LR against the null also exceeds the same threshold.

## Conclusion

This paper presents and discusses methods for DNA-based identification. Restricted approaches, in which the victims are considered separately or sequentially, may give inconsistent, ambiguous results. We therefore generally recommend the combined approach summarised by Algorithm 3. The idea is simple: first take care of the virtually obvious pairings, and then do a complete search to resolve the remaining. The resulting joint solution should be supplemented by posterior pairing and non-pairing probabilities, which summarise the evidence for each individual identification.

All methods described in this paper are implemented in the R package **dvir**, which is freely available from the official R repository (CRAN) and runs on all platforms. The documentation of the package provides further details and instructive examples.
